# Physicochemical Evaluation of the Upper Litani River Watershed, Lebanon

**DOI:** 10.1100/2012/462467

**Published:** 2012-04-30

**Authors:** Mark Saadeh, Lucy Semerjian, Nabil Amacha

**Affiliations:** ^1^Environmental Department, Associated Consulting Engineers, P.O. Box 11-3446, Beirut, Lebanon; ^2^Department of Civil and Environmental Engineering, Faculty of Engineering and Architecture, American University of Beirut, P.O. Box 11-0236, Beirut, Lebanon; ^3^Environmental Unit, Litani River Authority, P.O. Box 11-3732, Beirut, Lebanon

## Abstract

This study aims to determine the extent of groundwater damage in the Upper Litani River Basin (ULRB) after years of water mismanagement and overfertilization in what is considered to be Lebanon's largest fertile area. Physical and chemical samples were collected between 2005 and 2010 and analyzed using “The Standard Methods for the Examination of Water and Wastewater” (APHA, AWWA) in order to determine the extent of this pollution. The parameters included pH, ammonia, nitrate, nitrite, sulfate, phosphate, dissolved oxygen, and total dissolved solids.

## 1. Introduction

The issues of groundwater quality in Lebanon have become as important as quantity due to the rampant damage incurred by pollution sources, mainly agricultural and domestic. Overabstraction and withdrawal, as well as pollution are the orders of the day when it comes to groundwater resources of Lebanon whether along the coast or within the Bekaa Valley.

Lebanon's climate is characterized as being Mediterranean. In comparison to neighboring countries, Lebanon receives relatively higher amount of precipitation with an average of 823 mm/year, varying from 600 to 1,000 mm in the coastal area, 900 to 1,700 mm in the Lebanon mountain range, and 200 to 900 mm in the Bekaa Valley [1].

The objective of this paper is to evaluate the groundwater quality in the Upper Litani River Basin, with nearly 300,000 persons relying on it for their daily domestic needs as well as a complementary source for irrigation. The highest levels of contamination along the Litani River thus falls within the mid-Upper Litani River Basin, where the largest communities are located and are discharging their effluent into the river [2].

## 2. Study Area

The Litani River is Lebanon's longest and largest river flowing entirely within its boundaries. It originates in the Bekaa Valley from the Oleik spring (approximately 1,000 MASL) west of the ancient city of Baalbeck flowing southward then westward until it discharges into the Mediterranean Sea near the city Tyre. The entire trip covers nearly 170 km and ranges in flow between 14.2 m^3^/s during the wet season to about 4.4 m^3^/s in the dry season [3].

**Figure 1:**
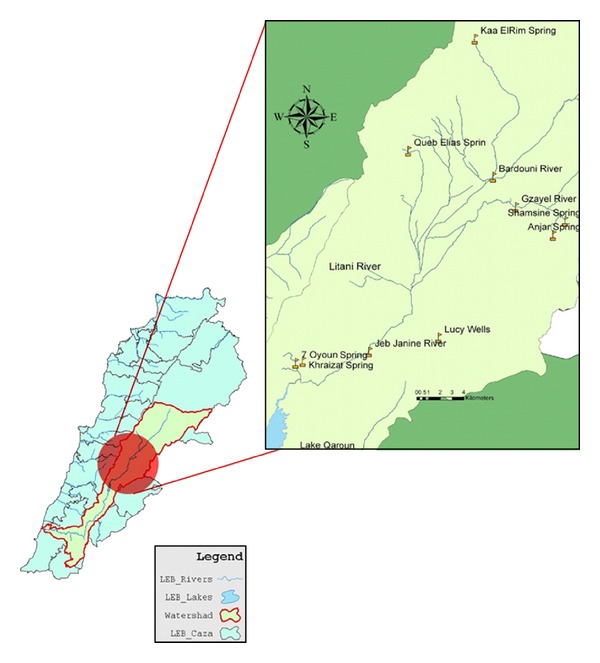
Sampling points in the upper Litani River Basin.

From a geomorphological perspective, the Litani River basin is divided into three sub-basins, with the largest being the Upper Basin stretching between its source until the Qaraoun dam at an elevation between 850 to 800 meters above sea-level (MASL) as depicted in Figure 1. The estimated area of this basin is approximately 1,400 km^2^, with several perennial tributaries feeding the Litani river, mainly Berdawni, Ghzayel, Qib Elias, and Chtoura. 

The Qaraoun reservoir can hold up to nearly 220 million cubic meters (MCM) of water with 160 MCM considered as active storage for irrigation and hydroelectricity. A study of isotope fractionation concludes that the Qaraoun reservoir is monomictic, meaning that it mixes from top to bottom once a year [5]. 

**Figure 2:**
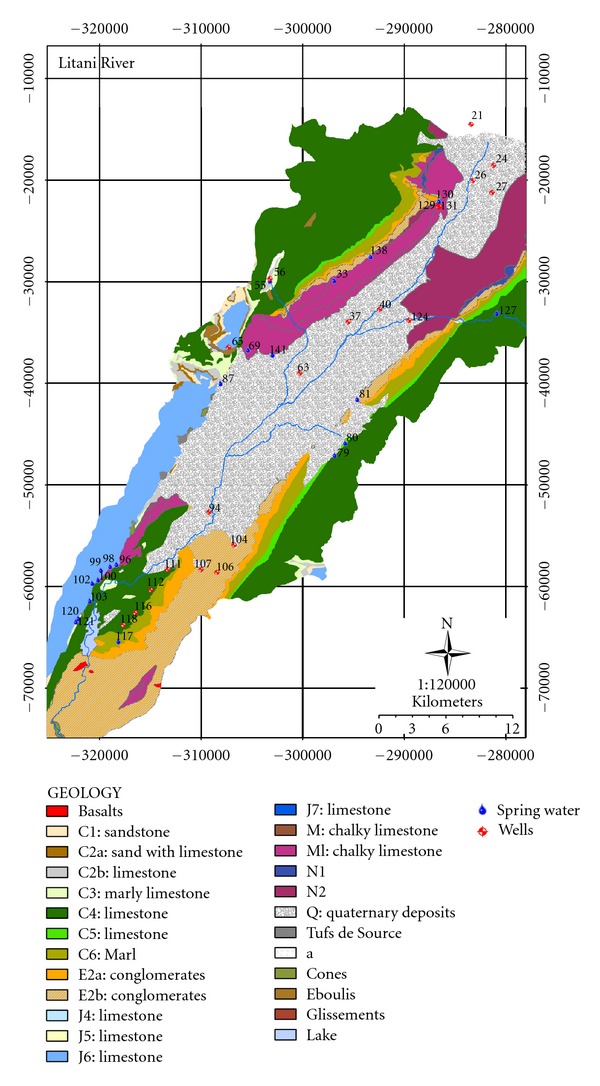
Geological formations of the Upper Litani River Basin [4].

The upper Basin is underlain by five aquifers as depicted in Figure 2. They include the following.

1. *Quaternary* (unconsolidated, semiaquiferous alluvial deposits found along the main Litani river course, q, alluviums).
2. *Neogene* (outcrop along the main Litani River course adjacent to the Quaternary deposits, m, conglomerate alluviums).
3. *Eocene* (marly limestone, semiaquiferous deposits that can reach a thickness of several hundred meters. They are mainly found in narrow outcrops running parallel on either side of the Litani River, e, marly limestones).
4. *Cenomanian* (considered to be one of Lebanon's major aquifers reaching a thickness up to 700 meters, C_4_, composed of dolomitic and marly limestone).
5. *Jurassic* (also considered to be another of Lebanon's major aquifers reaching a thickness up to several hundred meters. J_6_, composed of dolomitic limestone).

## 3. Methodology

Since 2005, the Environmental Unit of the Litani River Authority has been collecting and analyzing nearly 15 surface as well as groundwater samples from the entire Litani River Basin. Ten of these locations lie within the Upper Basin.

Sampling and measurement procedures were carried out in accordance with:

- D4449-01 Standard Guide for Sampling Groundwater Monitoring Wells [6],
- Groundwater well sampling [7].

The following physical and chemical parameters were selected based on previous studies conducted within the Upper Litani River Basin that had identified quite accurately sources of contamination. Analyzed parameters include pH, total dissolved solids (TDSs), dissolved oxygen (DO), ammonia, nitrate, nitrite, phosphate, and sulfate.

The various parameters were analyzed using standard procedures in accordance to “The Standard Methods for the Examination of Water and Wastewater” (APHA, AWWA). TDS, DO, and pH were measured on site using individually calibrated portable testers (LaMotte, USA).

For the remaining parameters, water samples were collected in polyethylene bottles, filtrated through a 0.45 *μ*m filter, and stored at 4°C. They were then analyzed spectrophotometrically with pertinent and certified reagents (LaMotte, USA). Finally, the results were evaluated in accordance to the Lebanese Standards Institution (LIBNOR) NL161: 1999, Standard for Drinking Water, the World Health Organization (WHO) Guidelines for Drinking Water Quality [9]; European Union (EU) Guidelines [8] as per Table 1.

As for the softwares used in this study, ArcGIS 9.3 was used in order to generate the geological as well as the hydrogeological maps. On the other hand, SURFER was used to generate the figures pertaining to the spatial distributions of the different parameters discussed in this study.

It is noteworthy to mention that Lebanon lacks any monitoring program of any scale for surface waters or ground waters, what exists in fact are studies that merely offer snapshots of the existing hydrochemical conditions, often lacking spatial and temporal variation. 

So for the purpose of this study, a myriad of sources were sampled including tributaries, springs, and a few public and private wells. In many of the wells of the Upper Litani River Basin, the water table has reportedly dropped more than 100 meters since the 1970s. The general direction of groundwater flow is from north to south.

## 4. Results and Discussion

### 4.1. Physicochemical Parameters

#### 4.1.1. pH

**Figure 3:**
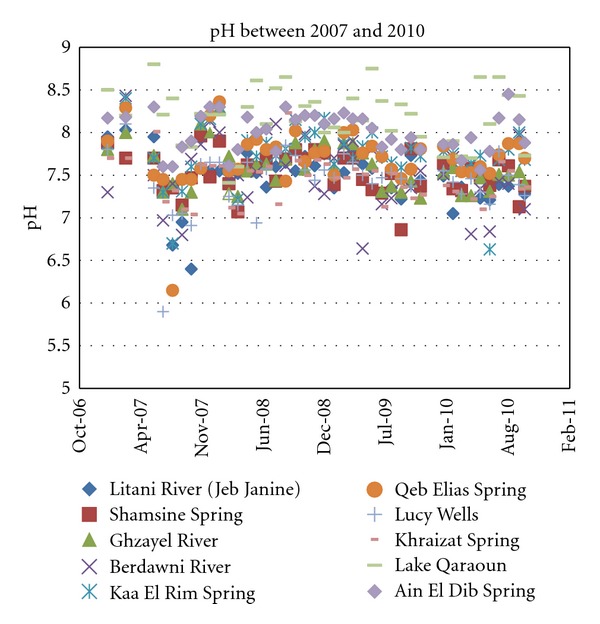
Recorded pH levels for years 2007 through 2010.

The pH values of the tributaries and groundwater as shown in Figure 3 are in the range of 6.5 to 8.5. Compared to the WHO guidelines for drinking water, the pH values for surface and groundwater fall within normal limits. It is noteworthy to mention that pH of the Qaraoun reservoir over the years has been the highest ranging between 8.0 and 8.5. This would indicate an alkaline water impoundment affected by the surrounding C_4_ (Sannine limestone) formation.

#### 4.1.2. Total Dissolved Solids

**Figure 4:**
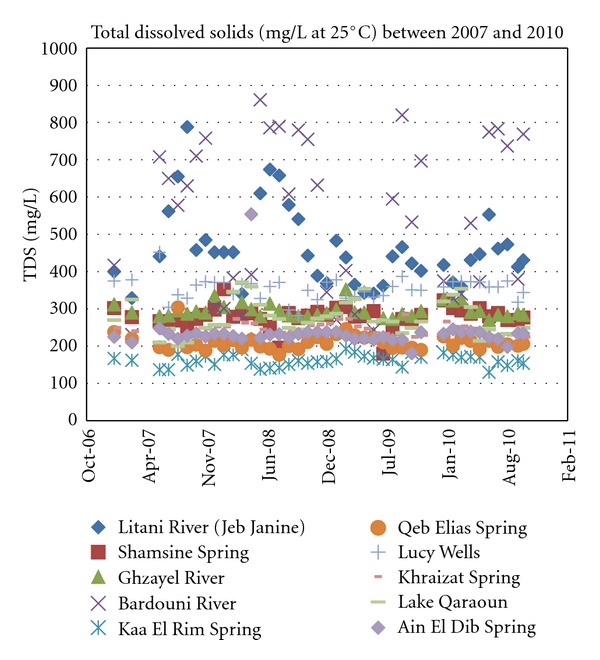
Recorded total dissolved Solids concentrations for years 2007 through 2010.

Most sources, whether surface or ground, have a TDS in the normal range between 100 and 500 mg/L. This range is acceptable for human consumption as well as irrigation purposes. The two sources that stand out with a relatively high TDS range are the Berdawni tributary and Litani river as shown in Figure 4. As for the former, the elevated levels of TDS would be explained by the fact that this tributary passes through the most populated urban center, Zahleh with an estimated population of more than 150,000 during the summer.

The high TDS range of the Litani river itself may be attributed to the fact that it receives runoff from all other tributaries including the heavily polluted Berdawni.

#### 4.1.3. Electrical Conductivity

**Figure 5:**
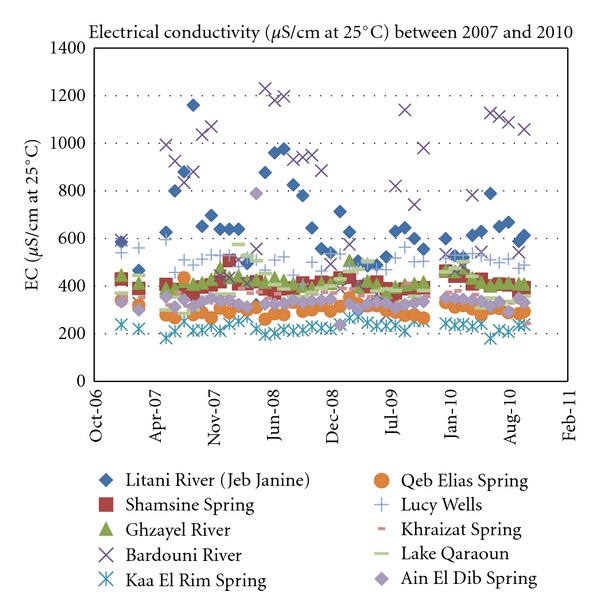
Electrical conductivity values for years 2007 through 2010.

Values for electrical conductivities are presented in Figure 5.

For comparison, EC values in *μ*S/cm were converted to chloride contents in mg/L using the following conventional formula:

(1) $${\text{Chloride}}_{\text{mg}/\text{L}}=0.14\times {\text{EC}}_{\mu \text{S}/\text{cm}}.$$

With Figure 5, it is apparent that chloride values for all the sampled sources, whether surface or subsurface, do not exceed 180 mg/L. In this chloride range, water is deemed suitable for most applications including drinking, domestic use, irrigation, and livestock.

#### 4.1.4. Dissolved Oxygen

**Figure 6:**
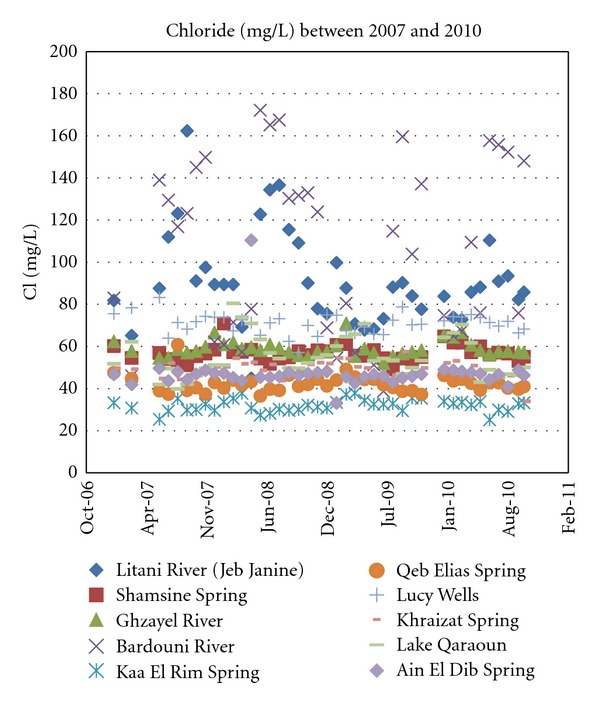
Chloride concentrations for years 2007 through 2010.

Again, most sources have a normal DO range as depicted in Figure 6, which would not place any undue stress on the biota of the tributaries, except for the Berdawni tributary and the Litani River. The levels of DO measured in the aforementioned, are dangerously low often dropping to 3 mg/L rendering them unsuitable for most fresh water species. It is noteworthy to mention that wastewater treatment in Lebanon as well as the Litani River Basin is practically nonexistent.

#### 4.1.5. Ammonia

Ammonia (NH_3_  or  NH_4_^+^) contamination is usually an indicator of sewage pollution which most certainly applies to Lebanon for its lack of a national wastewater treatment system. In some cases, ammonia in ground waters could reach as high as 3 mg/L [9]. In any case, ammonia has no drinking water standard when it comes to WHO or USEPA guidelines.

**Figure 7:**
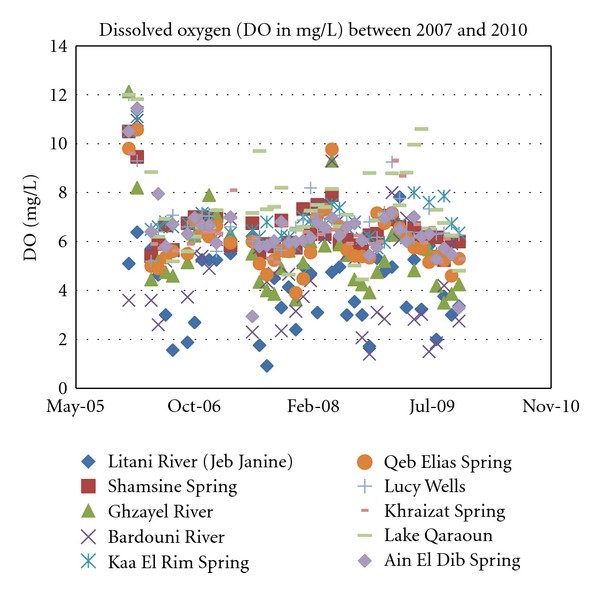
Dissolved oxygen levels for years 2007 through 2010.

As depicted in Figure 7, almost all sources have concentrations in the range of a few milligrams per liter consistent over the years. The only two exceptions are not surprisingly the Berdawni river and the Litani River. 

#### 4.1.6. Nitrate

Nitrate is considered to be very mobile due to its solubility and anionic form, moving long distances in water without transformation [10].

High concentrations of nitrate in groundwater is a cause for concern since it can lead to “blue baby syndrome” or methemoglobinemia if consumed by children under the age of six months. Additionally, studies indicate significant positive correlations between exposure to nitrate and cancer risk [10].

Normally, guidelines from around the world limit the acceptable amount of nitrates in drinking water to about 44 mg/L. Excessive amounts are usually attributed to intensive agricultural practices, which is certainly the case in the Upper Litani River Basin. The contribution from septic tanks and raw sewage to the nitrogen load in groundwaters cannot be overlooked.

**Figure 8:**
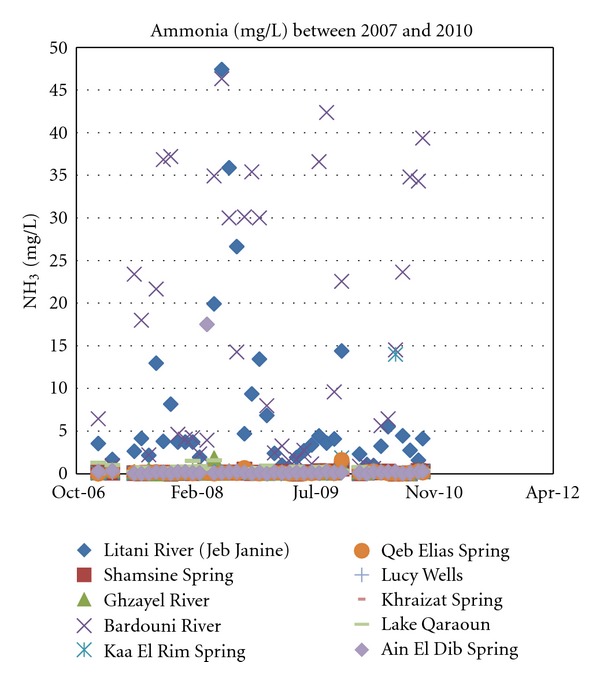
Ammonia levels for years 2007 through 2010.

In Figure 8, it is apparent that one source stands out over all the other monitored through the years in the Upper Litani River Basin, Lucy Wells. It so happens that these public wells were installed and are still being operated by local authorities for the supply of domestic water to neighboring villages. This source has consistently exceeded most standards set for nitrates in drinking water. This is testimony to the rampant discharge of raw sewage coupled with excessive agricultural runoff evident in the Upper Litani River Basin.

#### 4.1.7. Nitrite

**Figure 9:**
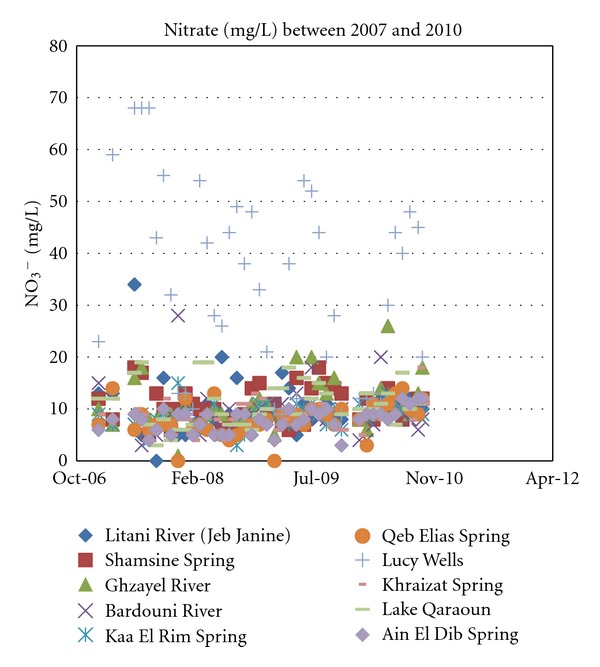
Nitrate concentrations for years 2007 through 2010.

Nitrite (NO_2_^−^) concentrations in all surface as well as groundwater sources of the study area were below most standards set for drinking water of 3.0 mg/L as shown in Figure 9. The presence of nitrite in groundwater is attributed to denitrifying bacteria occurring in anaerobic conditions but often in concentrations not exceeding a few milligrams per liter unlike nitrate.

#### 4.1.8. Phosphate

**Figure 10:**
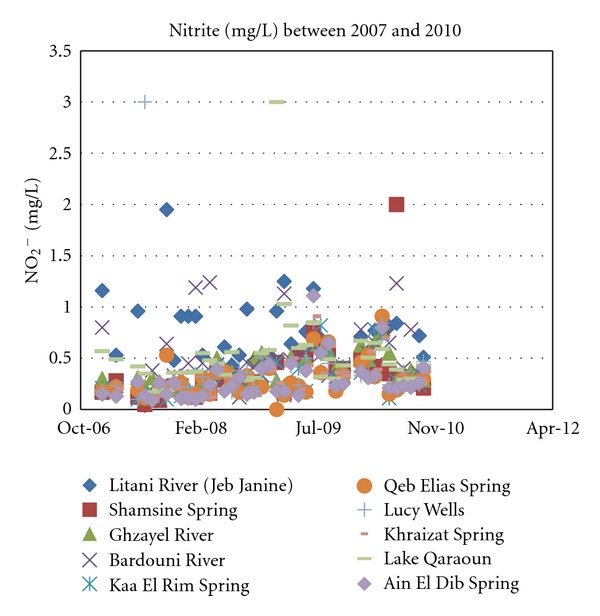
Nitrite concentrations for years 2007 through 2010.

Phosphorous in runoff from fertilized lands is usually low because the phosphate ions, having multiple negative charges, are bound strongly to mineral particles in soils [11]. This immobility is usually attributed to soil attenuation. Even with a large input of phosphorous in the Upper Litani River Basin from anthropogenic sources, namely, agricultural runoff, animal waste, raw sewage and household detergents, most sources rarely exceed a few milligrams per liter except for the Berdawni tributary and the Litani River (Figure 10).

**Figure 11:**
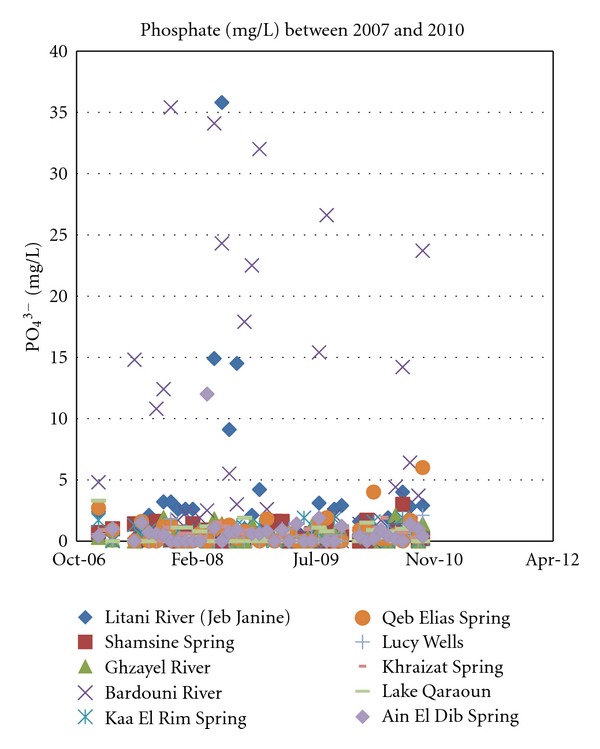
Phosphate concentrations for years 2007 through 2010.

Excess phosphate in surface runoff is always a cause for concern since it inevitably leads to what is known as “cultural eutrophication.” During eutrophication, phosphates in freshwater lead to a favorable condition for algae and weed growth, which ultimately brings about the rapid demise of an ecosystem through oxygen depletion (Figure 11).

#### 4.1.9. Sulfate

Sulfates (SO_4_^−2^) in water are usually attributed to atmospheric deposition, industrial runoff, and natural resources such as gypsum and anhydrite. Sulfate has no WHO health-based guideline; however USEPA has assigned a secondary contaminant limit (SMCL) of 250 mg/L.

**Figure 12:**
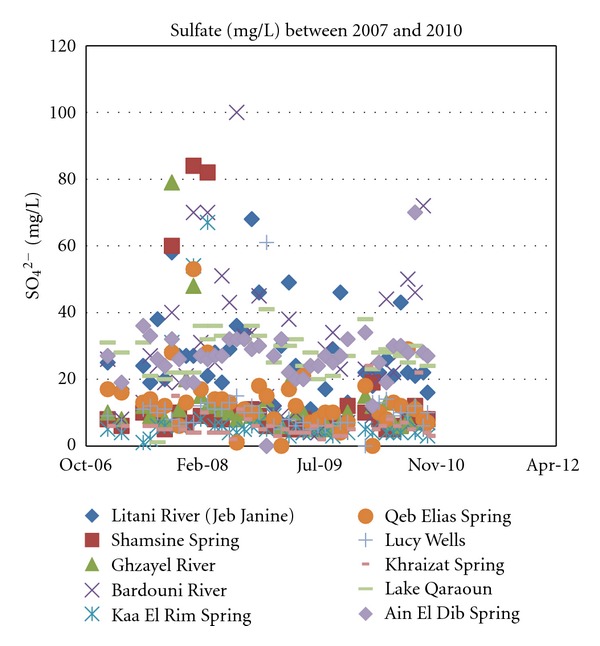
Sulphate concentrations for years 2007 through 2010.

In light of the following results, sulfate in surface and groundwater bodies have rarely exceeded 80 mg/L as shown in Figure 12 which is very unlikely to cause any health concerns.

### 4.2. Hydrochemical Facies

Major cations and anions can be plotted on a trilinear diagram also known as a Piper diagram. These ions include calcium, magnesium, sodium, potassium, chloride, sulfate, and bicarbonate. Since some of these ions were not included in the study, the Piper diagram cannot be utilized, but comparisons will be drawn for the analytes that were measured, namely, chloride, phosphate, sulfate, ammonium, nitrite, and nitrate.

In almost all sources, chlorides outrank all the other analytes in concentration, followed by nitrates in second place, phosphates, and ammonium drawing in third place, and nitrites in last place. As such, major concerns in the Upper Litani River Basin include salinity as well as eutrophication. Blue baby syndrome is also a major cause for alarm if the groundwater is consumed without any treatment to lower nitrates to safe levels.

### 4.3. Water Quality for Irrigation

So long as subsurface water is used reasonably for irrigation, there seems to be little danger of salinization. However, over abstraction (or over pumping) is rampant in the Upper Litani River Basin, as such; the water table is lowering in fact several meters per year [12].

For irrigation purposes, guidelines are less stringent than for those of drinking water. Accordingly, salinity of water, expressed in *μ*S/cm, is suitable for irrigation so long as it does not exceed 750 *μ*S/cm [13]. Referring to Figure 5, most sampled waters have salinity below the 750 *μ*S/cm guideline throughout the year, and so are suitable for irrigation in the Upper Bekaa Valley.

Lastly, if subsurface water is used in modicum for irrigation purposes, there may not be any need to add fertilizers in the coming years, for the aquifers are quite saturated with nitrates and phosphates as it is. As for the use of the Litani river and its tributaries for irrigation, extreme caution should be observed for their waters are laden with pollutants typically found in raw sewage.

## 5. Conclusions

This study is a culmination of nearly five years of continuous monitoring in the Upper Litani River Basin, offering a unique insight into the general anthropogenic impacts on the surface as well as subsurface water conditions. The main concern comes from nitrates and phosphates which have leached into underlying aquifers well beyond permissible concentrations intended for human consumption. Furthermore, even higher concentrations of said parameters in surface waters has led to severe eutrophication of the Litani river, as well as the Qaraoun reservoir, entailing the use of copper sulfate on a continual basis by the local authorities to keep algae and weeds in check.

As for the remaining parameters, chloride, sulfate, pH, EC, and DO, their concentrations are usually within permissible standards set for human consumption, irrigation and river quality indicators.

A Water Evaluation and Planning (WEAP) simulation model shows that the current practice of discharging untreated sewage into the river system is already causing a wide-scale pollution that escalates to an alarmingly hazardous state during drier times, which last for the longer part of the year, and possibly for several years in a row during drought spells [14].

Accordingly, it is imperative that a national monitoring plan for Lebanon's rivers and aquifers be finally implemented in order to combat pollution and water scarcity in a region that is expected to suffer the brunt of global warming. As a first step, the Litani River Authority should be afforded all means to make the transition into a river basin agency in charge of Lebanon's most important lotic system.

## Figures and Tables

**Table 1:** Comparison of various drinking water guidelines.

Parameters	LIBNOR	EU standards	WHO standards
pH	6.5–8.5	6.5–9.0	No guideline
Total Dissolved Solids (mg/L)	500	No guideline	No guideline
Electrical Conductivity (*μ*S/cm at 20°C)	No guideline	2,500	No guideline
Chloride (mg/L)	200	250	No guideline
Dissolved Oxygen (mg/L)	No guideline	No guideline	No guideline
Ammonia (mg/L)	No guideline	0.5	No guideline
Nitrate (mg/L)	45	50	50
Nitrite (mg/L)	0.05	0.5	3.0
Phosphate (mg/L)	1.35	No guideline	5.0
Sulphate (mg/L)	250	250	500
